# Supplementary material for: Geometric isotope effect of deuteration in a hydrogen-bonded host–guest crystal
Source: Nat Commun. 2018 Feb 2;9:481. doi: 10.1038/s41467-018-02931-8 (PMC5797174; doi:10.1038/s41467-018-02931-8)
Supplement: Supplementary file 1 — Supplementary Information [file 41467_2018_2931_MOESM1_ESM.pdf]

Supplementary Information

Geometric isotope effect of deuteration in a hydrogen-bonded host–guest crystal

Shi et al.

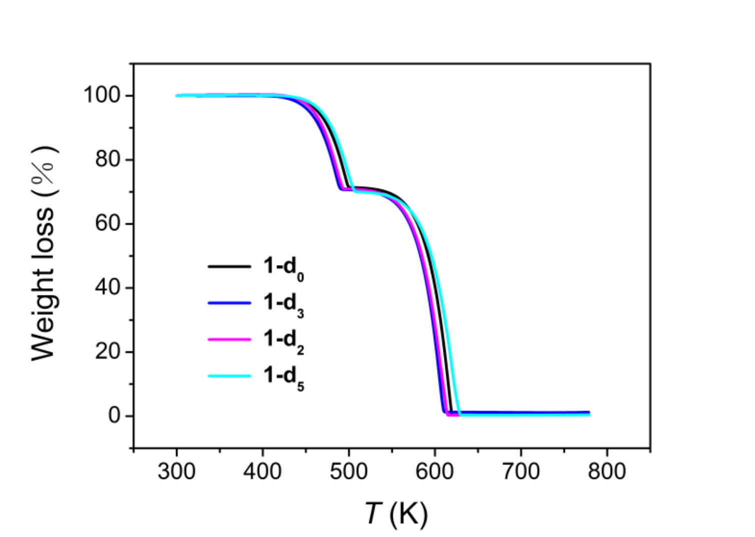

Supplementary Figure 1 | TGA measurements of 1-d$_0$−1-d$_5$. The decomposition begins at about 430 K.

a

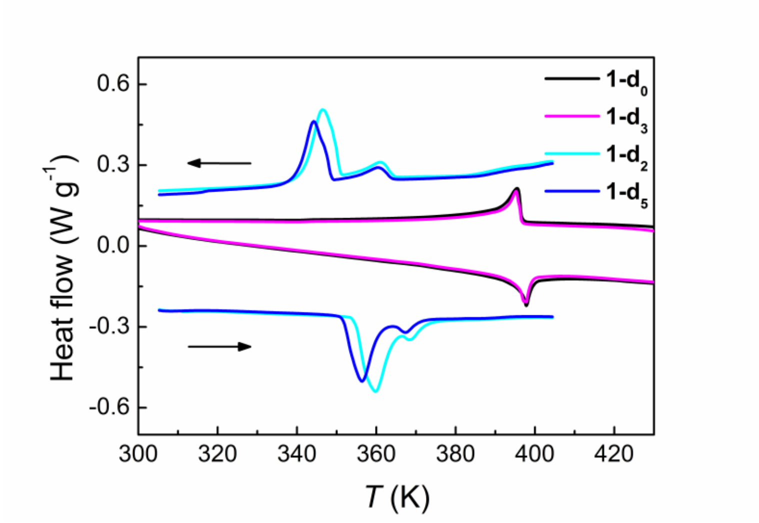
1-d0
1-d3
1-d2
1-d5
Heat flow (W g-1)
T (K)
0.6
0.3
0.0
-0.3
-0.6
300
320
340
360
380
400
420

b

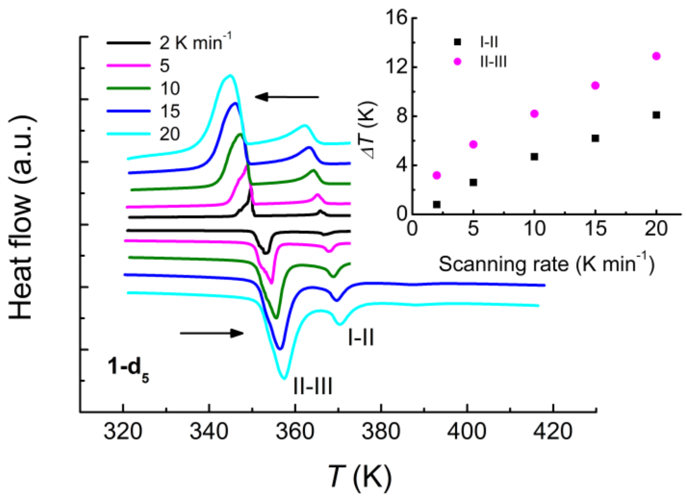
2 K min-1
5
10
15
20
1-d5
I-II
II-III
Heat flow (a.u.)
T (K)
320
340
360
380
400
420
I-II
II-III
ΔT (K)
Scanning rate (K min-1)
0
5
10
15
20
0
4
8
12
16

c

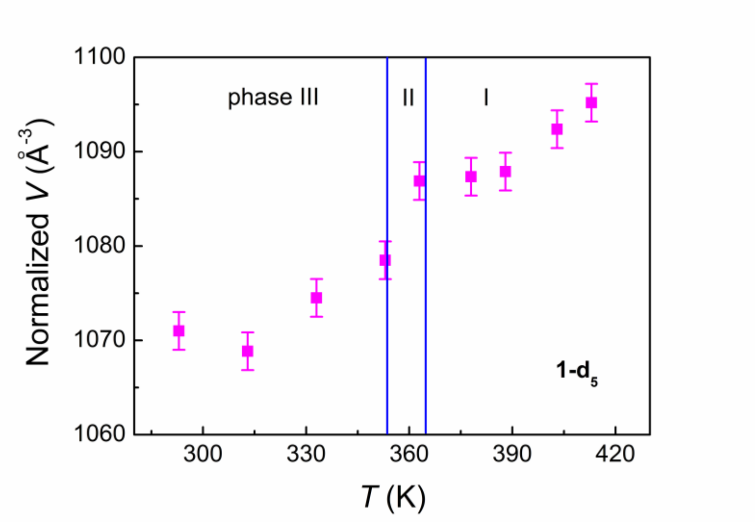

Supplementary Figure 2 | Characterizations of the phase transitions. (**a**) DSC curves of **1-d$_0$−1-d$_5$** obtained in a heating-cooling cycle. (**b**) DSC curves of **1-d$_5$** measured at different scanning rates. Inset: difference of peak temperature Δ*T* versus scanning rate. (**c**) Normalized unit cell volume of **1-d$_5$** versus temperature. The error bar is the estimated s.d. Note: (**b**) and (**c**) show the characteristics (first or second order) of the I-II and II-III phase transitions.

a

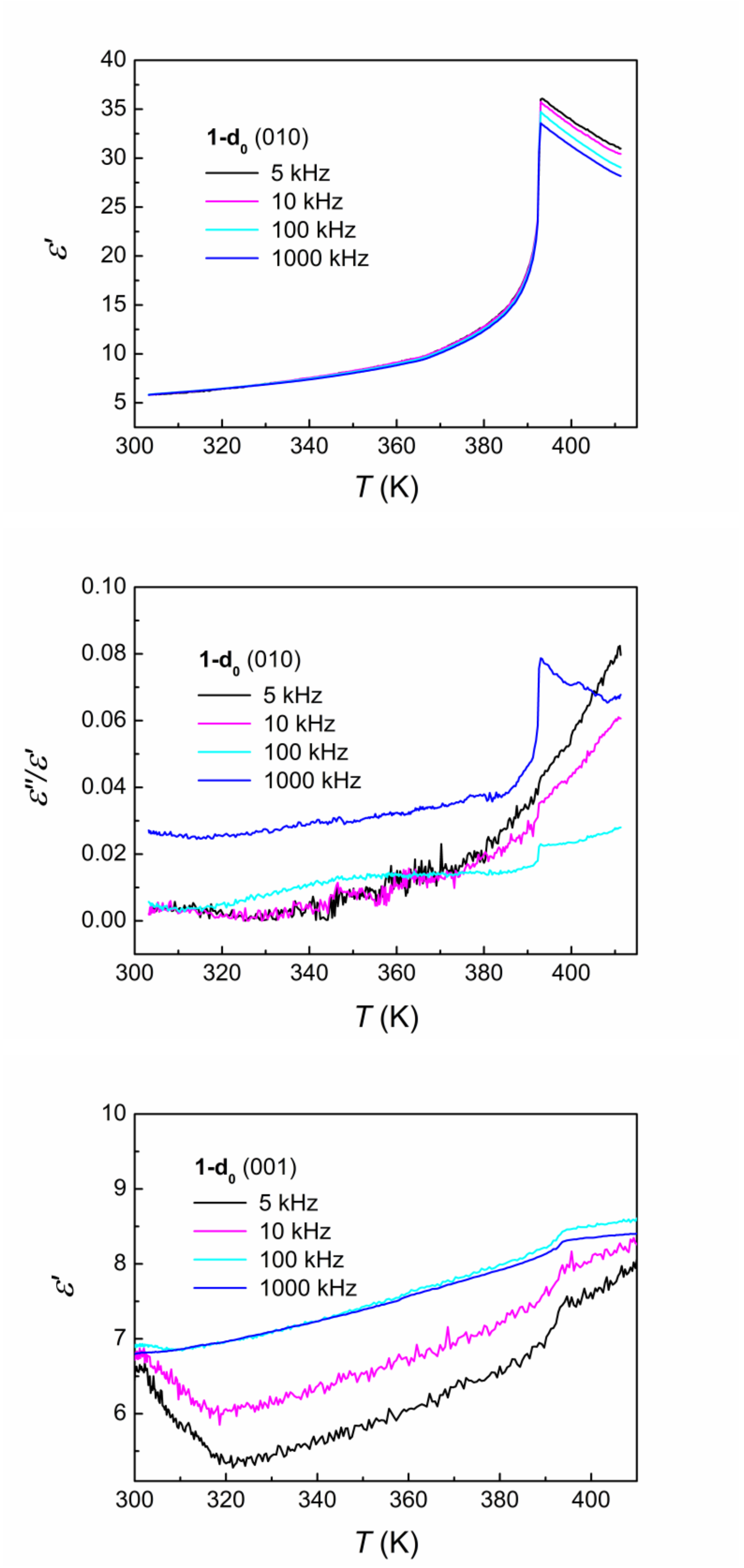

1-d0 (010)
5 kHz
10 kHz
100 kHz
1000 kHz
ε'
T (K)
1-d0 (010)
5 kHz
10 kHz
100 kHz
1000 kHz
ε''/ε'
T (K)
1-d0 (001)
5 kHz
10 kHz
100 kHz
1000 kHz
ε'
T (K)

b

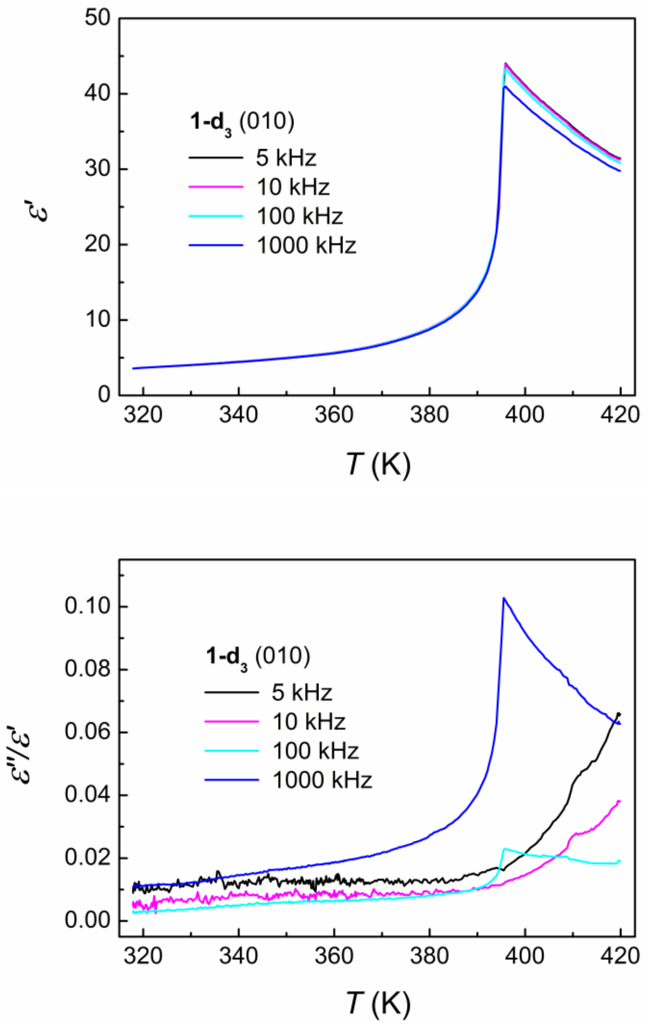

50
40
30
20
10
0
ε′
320
340
360
380
400
420
T (K)
1-d3 (010)
5 kHz
10 kHz
100 kHz
1000 kHz
0.10
0.08
0.06
0.04
0.02
0.00
ε″/ε′
320
340
360
380
400
420
T (K)
1-d3 (010)
5 kHz
10 kHz
100 kHz
1000 kHz

c

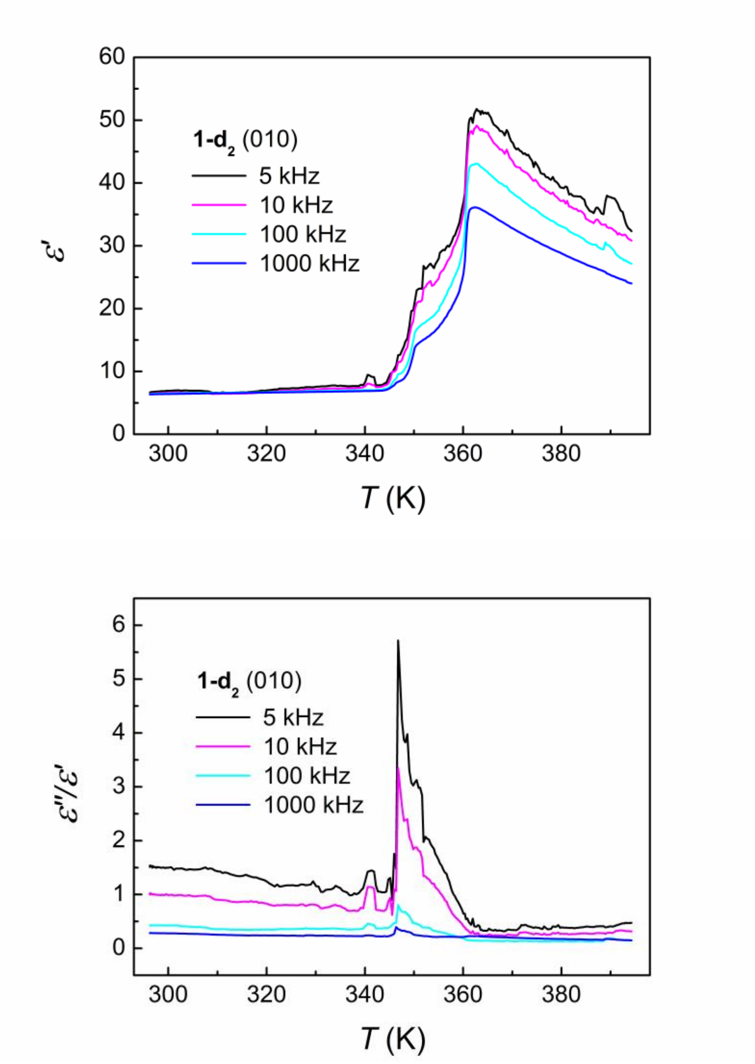

d

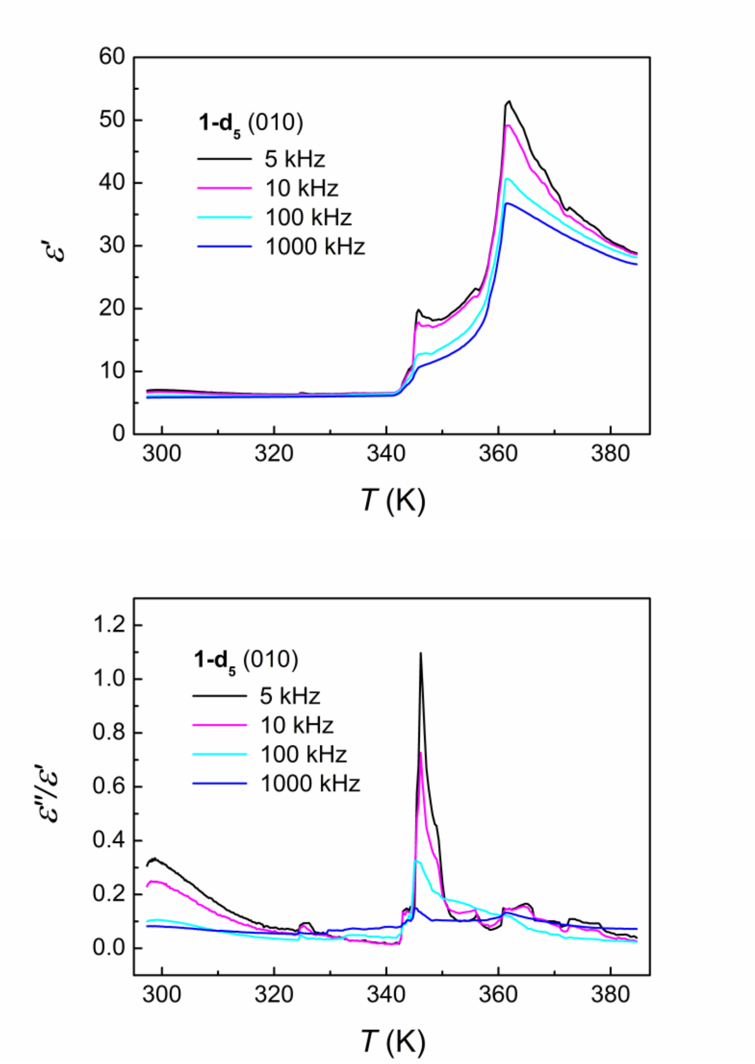

Supplementary Figure 3 | Dielectric spectra. Temperature dependence of the real part of dielectric constant (ε') and tangent loss ($\varepsilon''/\varepsilon'$) at different frequencies of 5−1000 kHz in a cooling mode: (**a**) **1-d$_0$**; (**b**) **1-d$_3$**; (**c**) **1-d$_2$** and (**d**) **1-d$_5$**. Note: all the directions are based on the structures at 413 K in phase I.

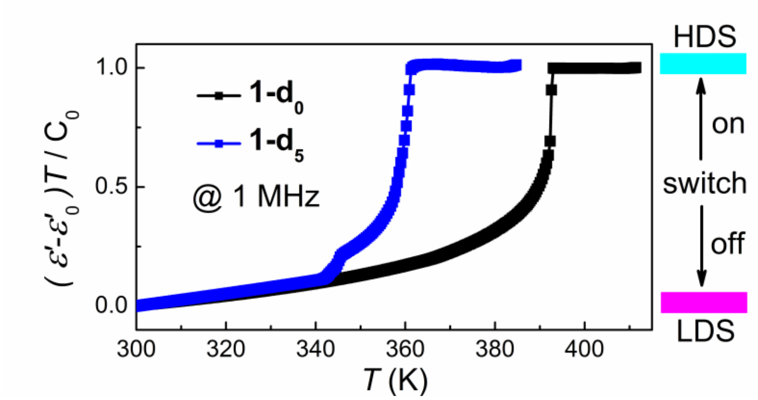

Supplementary Figure 4 | Dielectric transition. Normalized (ε'–ε'_0)T of **1-d$_0$** and **1-d$_5$** (the ε'_0 is the ε' in the LDS such as 300 K, assumed to be constant), measured on crystal plates upon cooling, showing a dielectric switching between LDS (off) and HDS (on). The largest dielectric responses appear on the (010) crystal plane which is a cleavage plane and designated according to the crystal structures at 413 K in phase I.

293 K

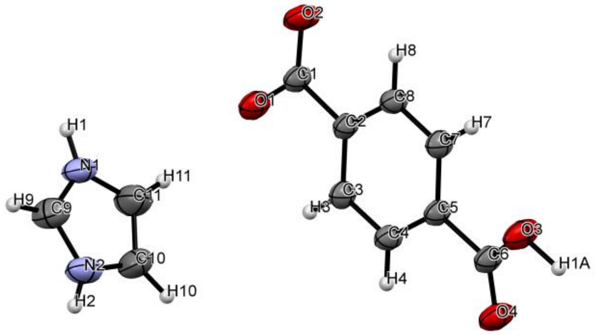
O2
H8
C1
C8
O1
C2
H7
H1
C7
N1
H11
C11
H9
C9
C3
C5
H3
C4
O3
C6
H1A
N2
C10
H2
H10
H4
O4

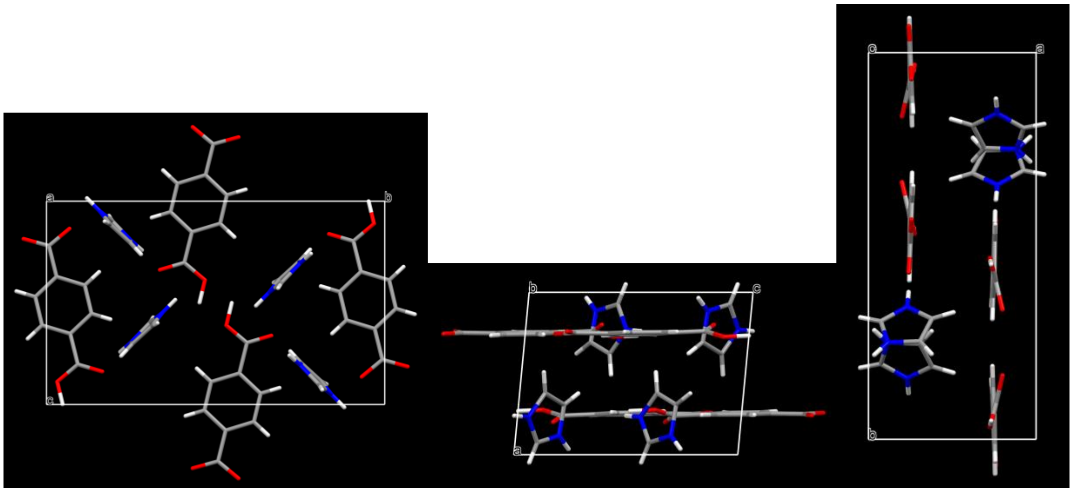

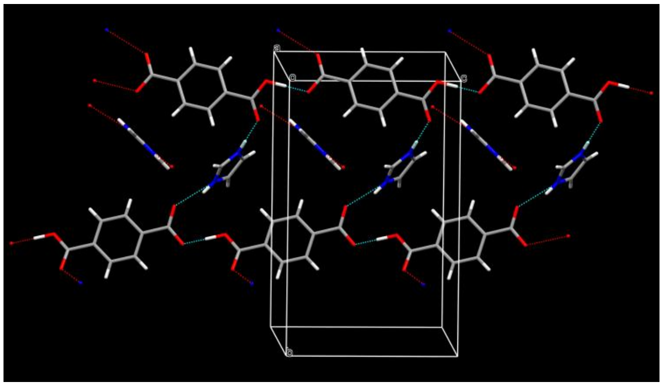

413 K

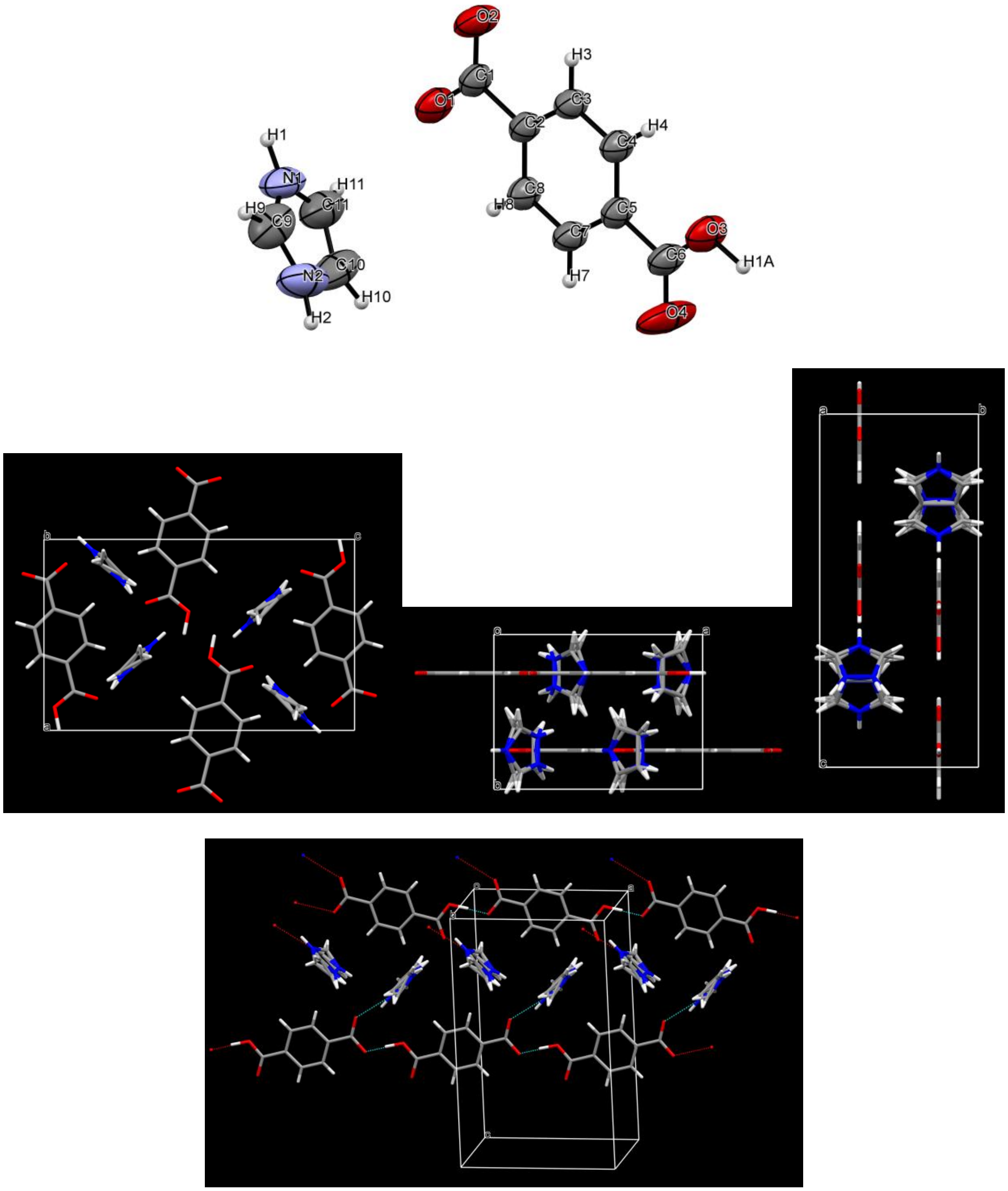

Supplementary Figure 5 | Crystal structure diagrams. Asymmetric units with thermal ellipsoids at 50% probability, crystal packings and H-bonding patterns of the H isomorph **1-d$_0$** at 293 K and 413 K. Dotted lines represent H bonds.

1-d_0 at 293 K

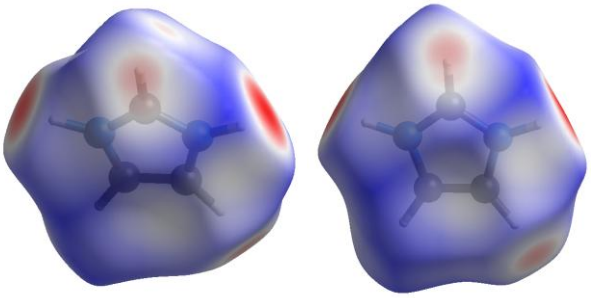

1-d_0 at 413 K

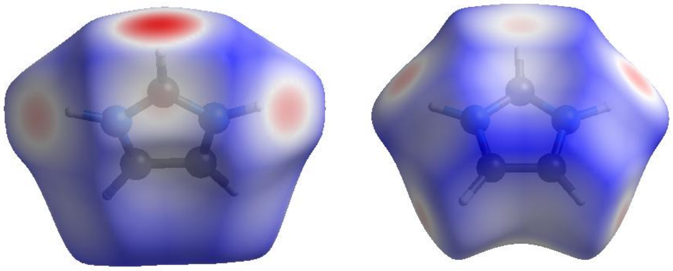

1-d_5 at 293 K

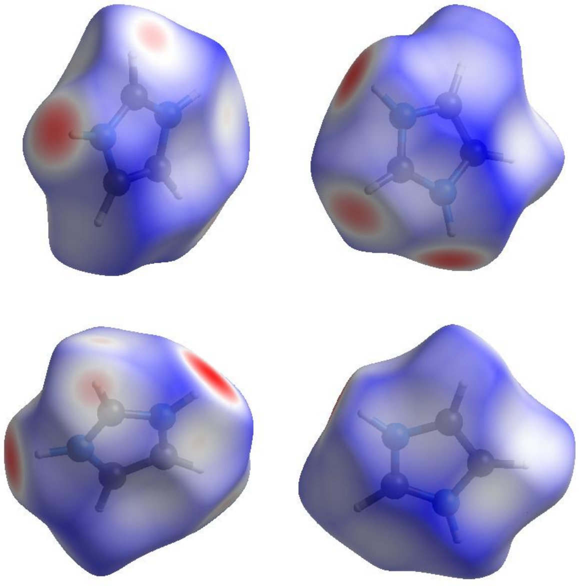

1-d_5 at 363 K

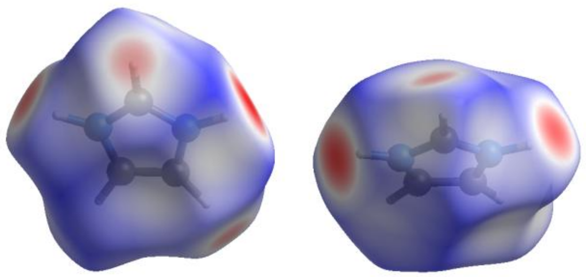

1-d_5 at 413 K

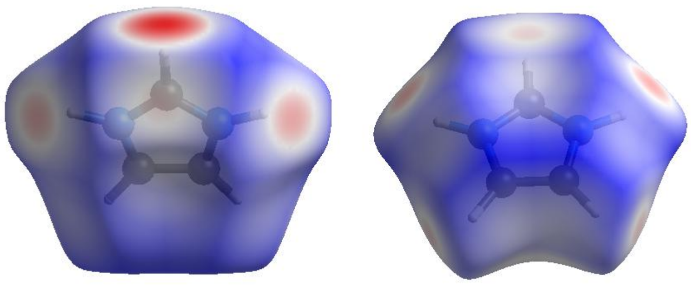

Supplementary Figure 6 | Hirshfeld surfaces. Front and back views of the Hirshfeld surfaces of the Im cations in **1-d_0** and **1-d_5** in different phases. The red, white and blue regions of the surfaces correspond to positive (close contact), neutral and negative isoenergy, respectively. The Im cation in phase I is shown in one of the two orientations.

293 K

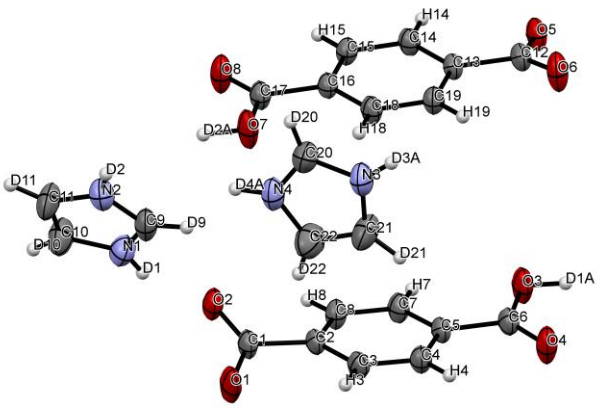
H14
H15
C15
C14
O5
O8
C13
C12
O6
C17
C16
C19
C18
H19
D20
D2A
O7
H18
C20
D3A
N3
D2
D11
N2
D4A
N4
C11
C9
D9
C10
C21
D10
N1
C22
D21
D22
D1
H7
O3
D1A
H8
C7
O2
C8
C6
C5
C1
C2
O4
C3
C4
H4
H3
O1

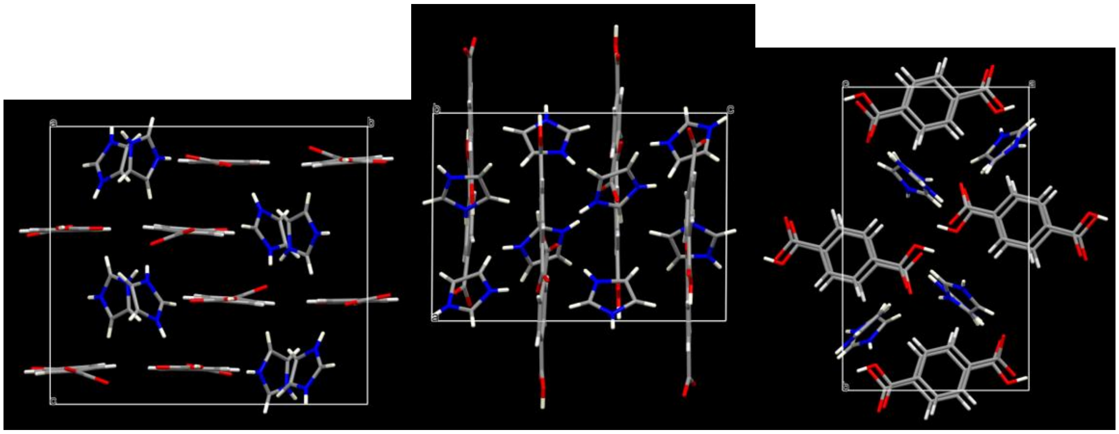

363 K

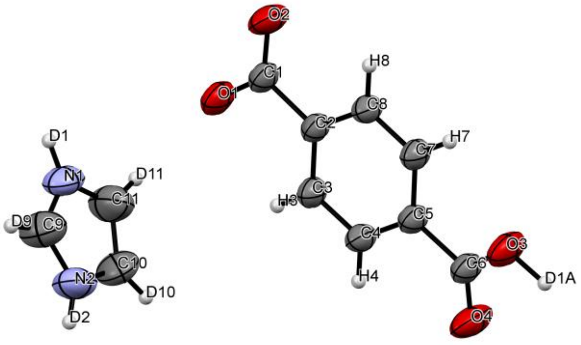

O2
C1
O1
H8
C8
C2
H7
C7
C3
H3
C5
C4
O3
C6
D1A
H4
O4
D1
N1
D11
C11
D9
C9
C10
N2
D10
D2

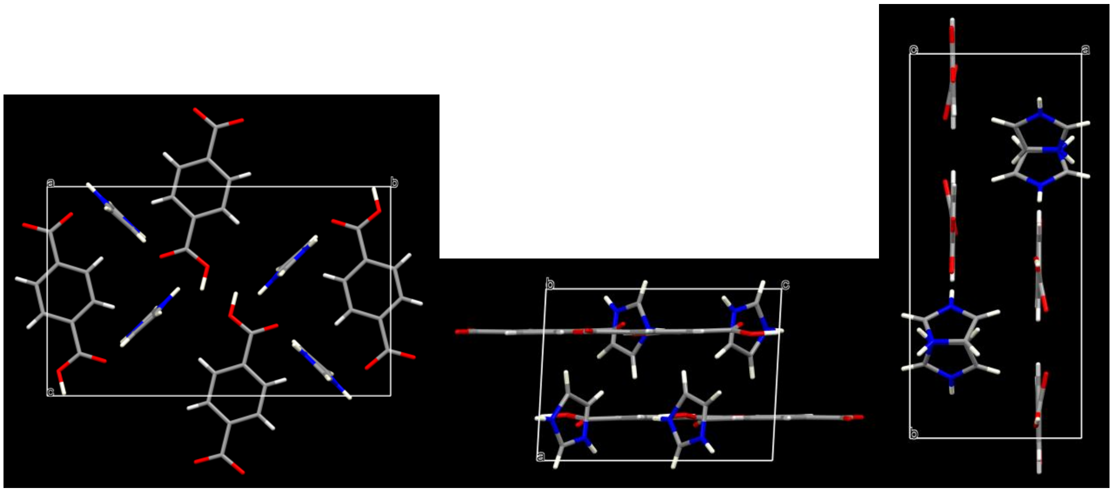

413 K

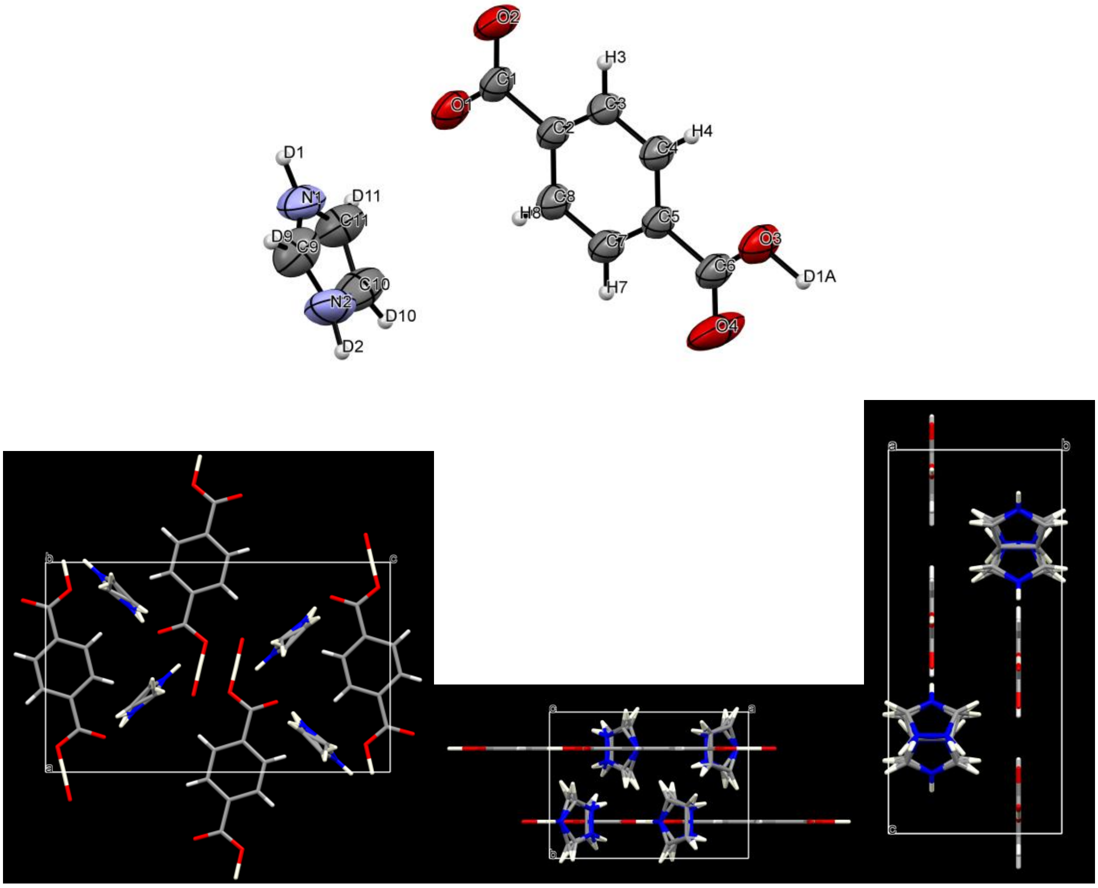

Supplementary Figure 7 | Crystal structure diagrams. Asymmetric units with thermal ellipsoids at 50% probability and crystal packings of the D isomorph **1-d$_5$** at 293 K, 363 K and 413 K.

293 K

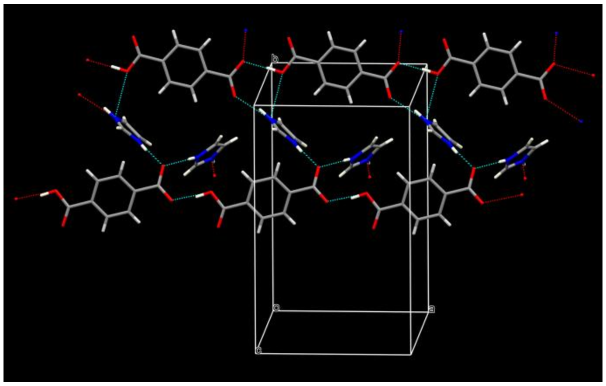

363 K

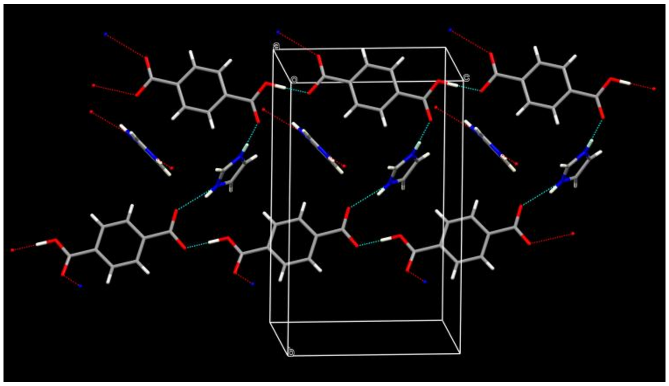

413 K

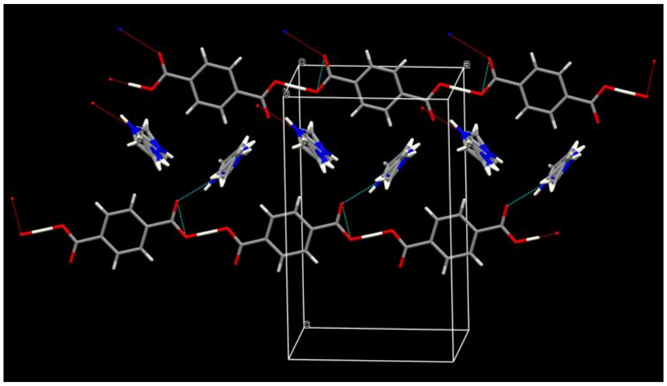

Supplementary Figure 8 | H-bonding patterns. The D isomorph **1-d$_5$** shown at 293 K, 363 K and 413 K. Dotted lines represent H bonds.

1-d_0 at 293 K

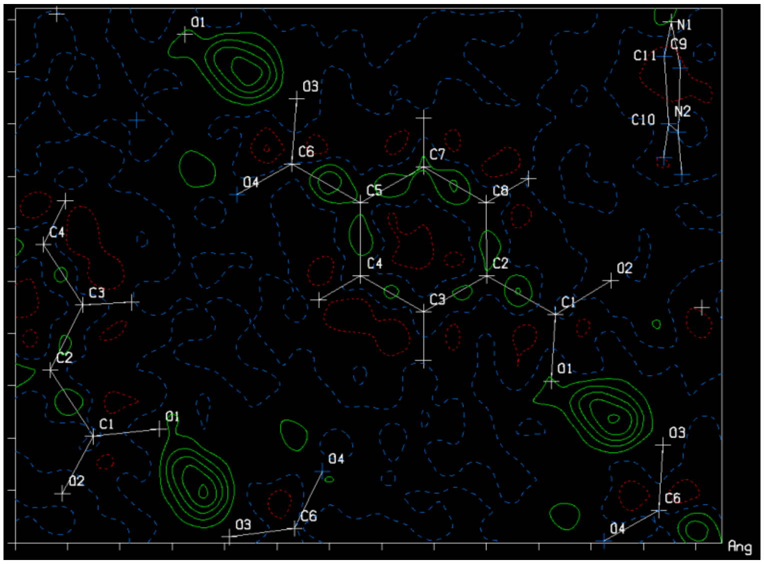

1-d_0 at 413 K

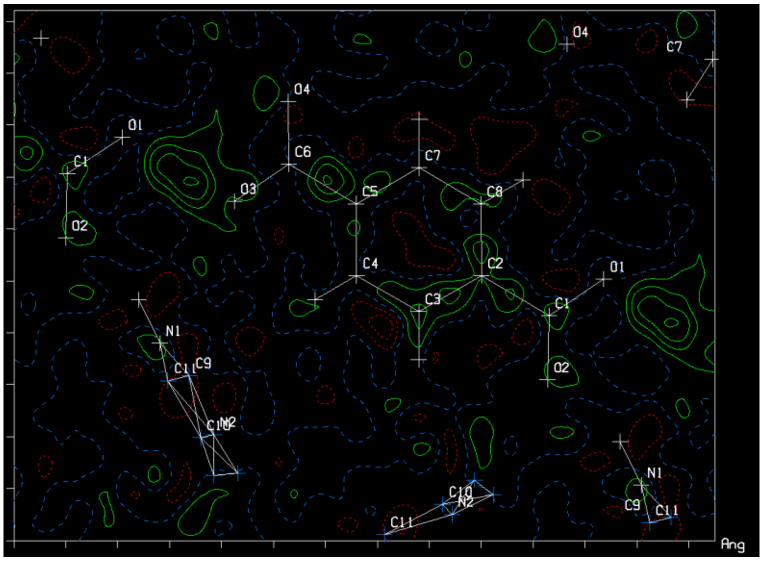

1-d_5 at 293 K

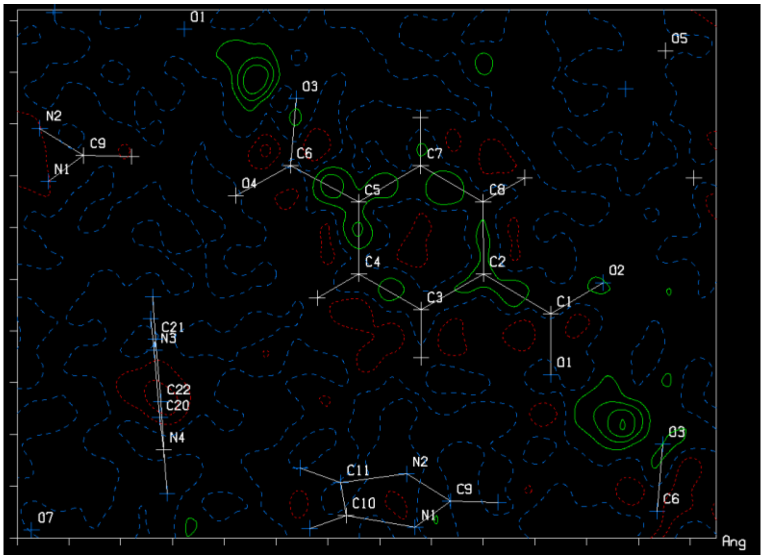
O1
O5
O3
N2
C9
N1
C6
O4
C7
C5
C8
C4
C2
O2
C3
C1
C21
N3
O1
C22
C20
N4
O3
N2
C11
C9
C10
N1
C6
O7
Ang

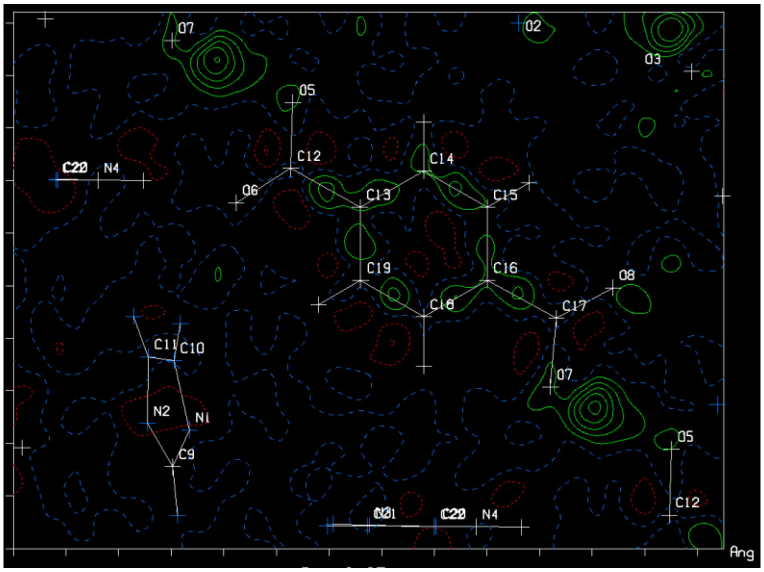
O7
O2
O3
O5
C12
C14
C20
N4
O6
C13
C15
C19
C16
O8
C18
C17
C11
C10
O7
N2
N1
O5
C9
C12
C21
C20
N4
Ang

1-d_5 at 413 K

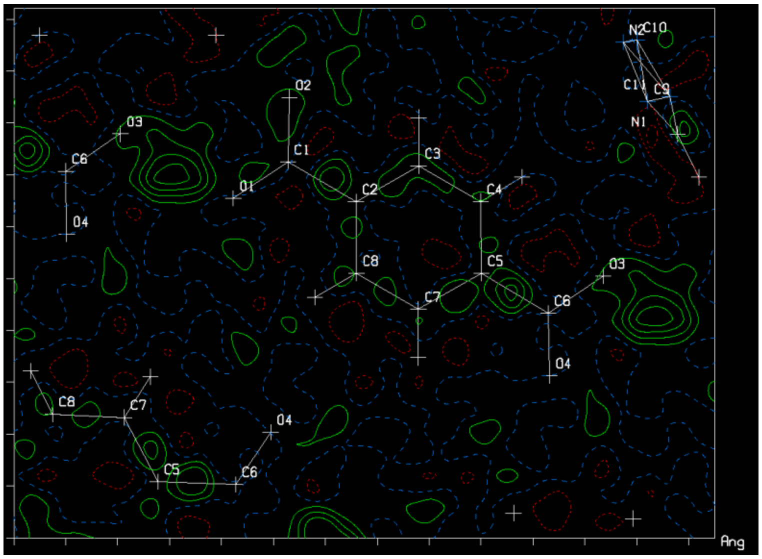

Supplementary Figure 9 | Difference Fourier maps. Plots of **1-d_0** and **1-d_5** visualized at 293 K and 413 K.

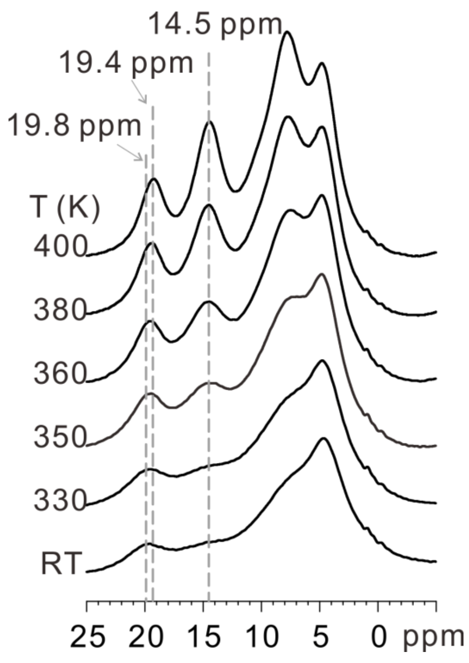

Supplementary Figure 10 | ^{1}H NMR of 1-d$_0$ in the temperature range 298−400 K.

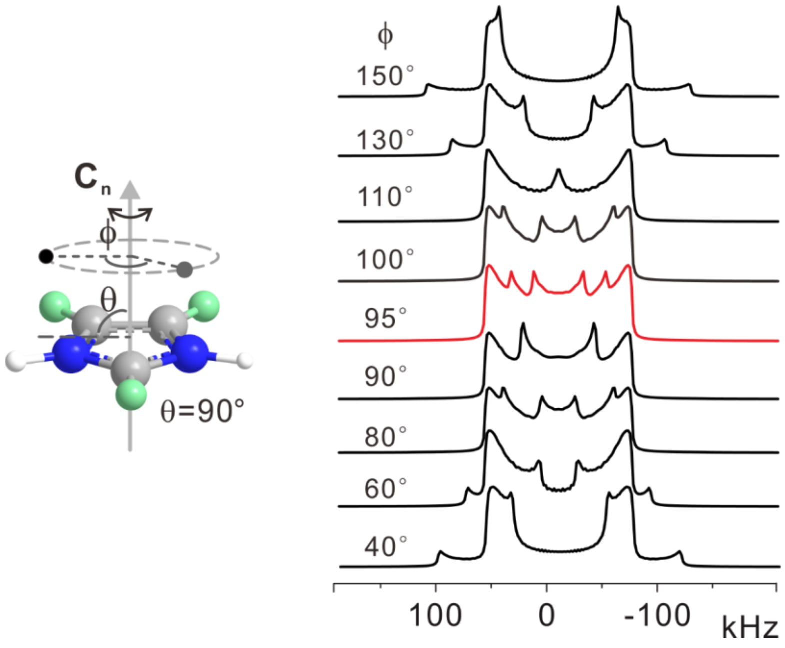

Supplementary Figure 11 | Simulated ^{2}H patterns for determination of the jump angle *ϕ* (i.e., the reorientation angle). The Im cation is assumed to have an in-plane 2-site jump motion. The quadrupole coupling used in the simulation is 134 kHz and the asymmetric parameter η = 0. The jump rate (i.e., the reorientation rate) is 300 kHz and assumed to have a lg(Gaussian) distribution with a standard deviation of σ = 1.

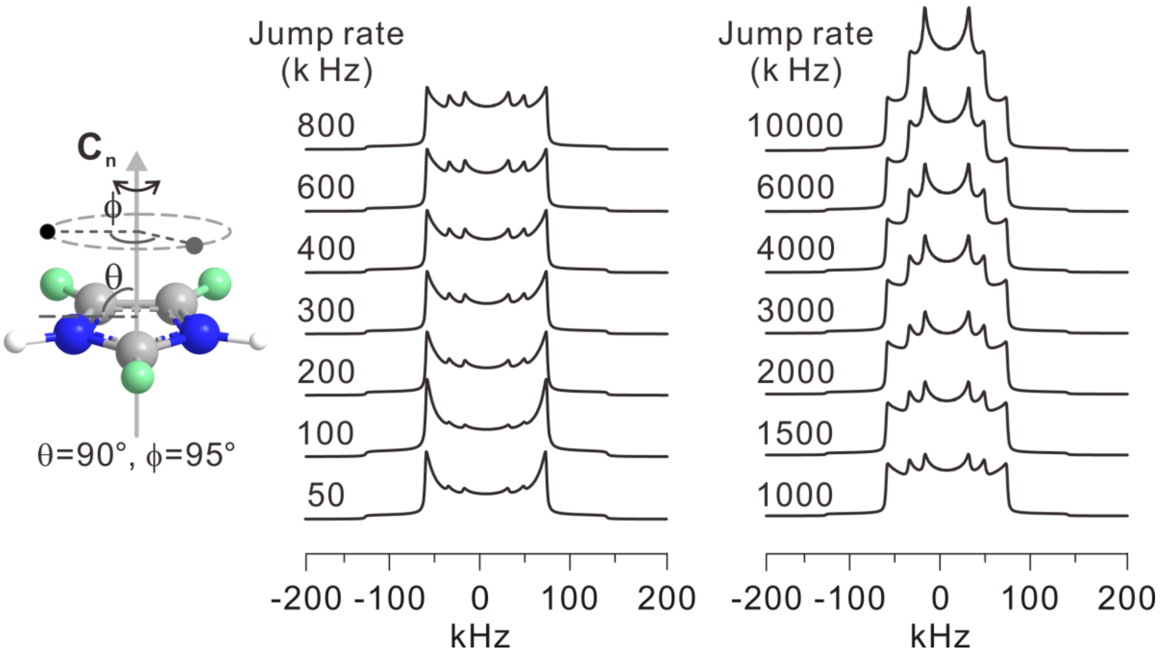

Supplementary Figure 12 | Simulated ^{2}H patterns for determination of the jump rate (i.e., the reorientation rate). The Im cation is assumed to have in-plane 2-site jump motion. The jump angle (i.e., the reorientation angle) is ϕ = 95°. The jump rate varies from 50 k to 10 M Hz and is assumed to have a lg(Gaussian) distribution with a standard deviation of σ = 1. A rigid component has been added in the simulation. The content of the rigid component is 15% for the patterns having jump rates ranging from 50 – 800 kHz, and 10% for the patterns having jump rates ranging from 1000 – 10000 kHz. The quadrupole coupling used in the simulation is 134 kHz and the asymmetric parameter η = 0.

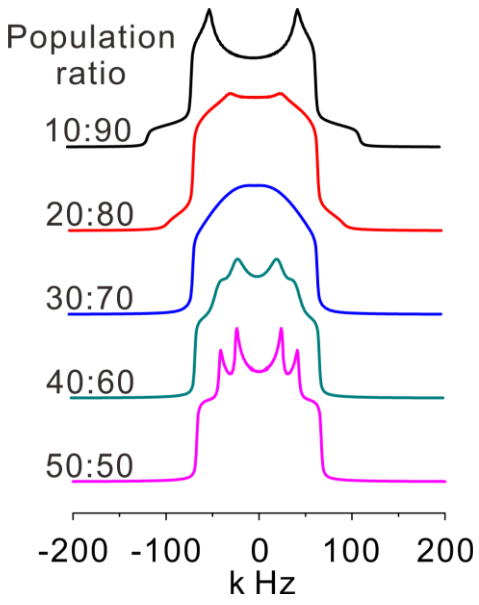

Supplementary Figure 13 | Simulated ^{2}H patterns showing the influence of the population ratio in the two sites on the line shape. The population ratio varies from 50:50 to 10:90. The Im cation is assumed to have an in-plane 2-site jump motion. The quadrupole coupling used in the simulation is 134 kHz and the asymmetric parameter η = 0. The jump rate (i.e., the reorientation rate) is 2000 kHz.

a

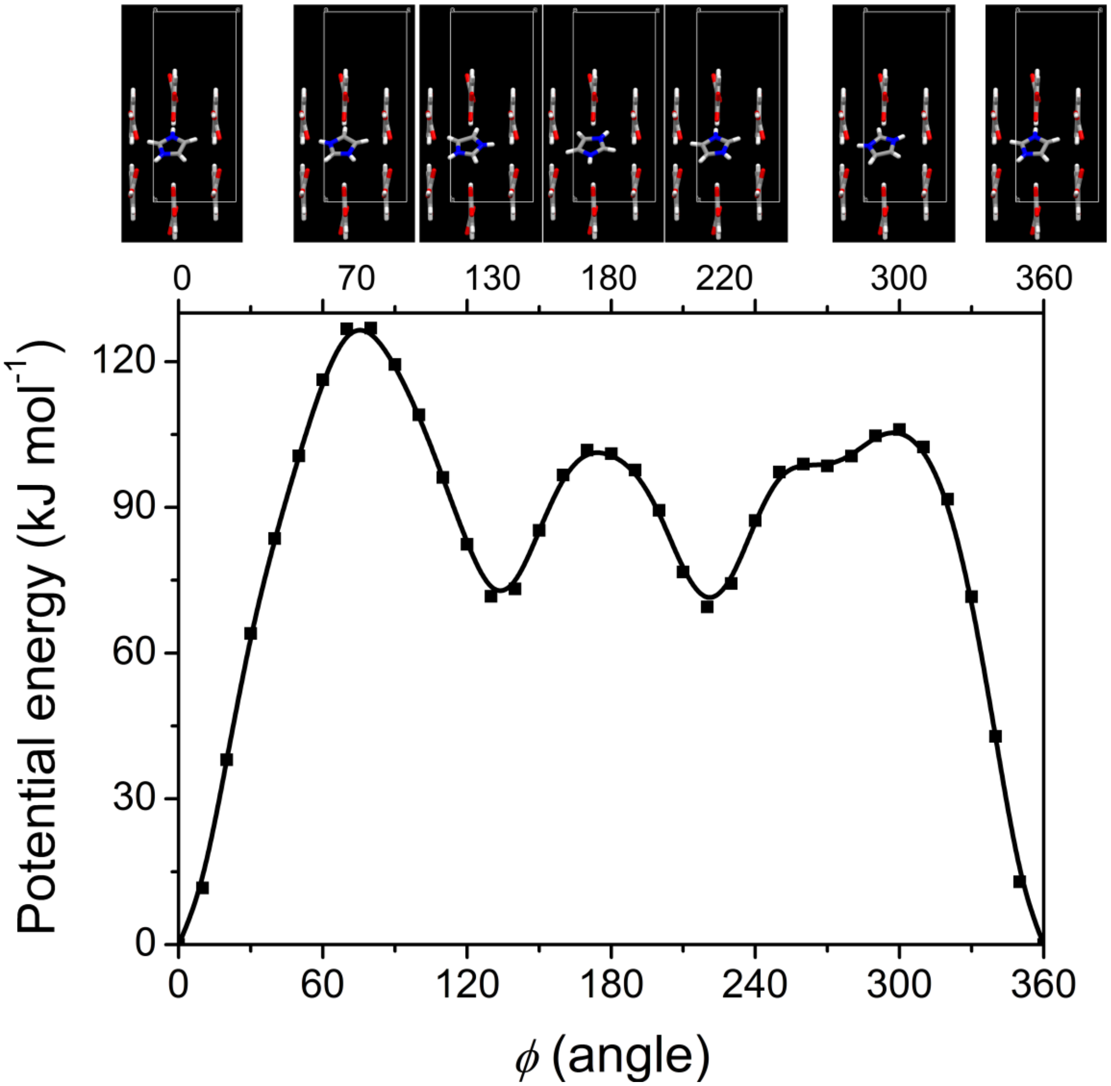
0
70
130
180
220
300
360
Potential energy (kJ mol-1)
120
90
60
30
0
0
60
120
180
240
300
360
ϕ (angle)

b

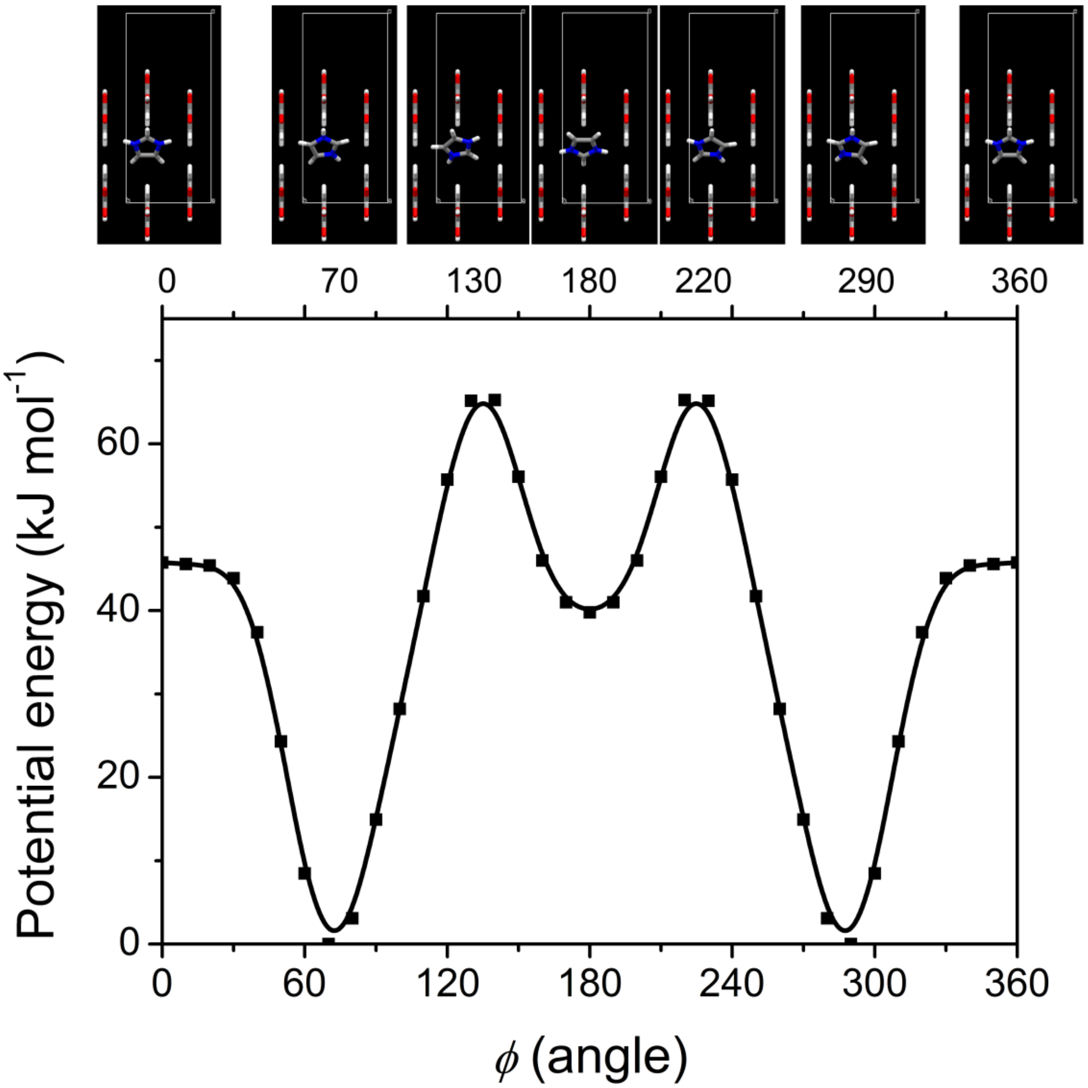

Supplementary Figure 14 | Rotational potential energy profiles. Plots of **1-d$_0$** at (**a**) 293 K (phase II) and (**b**) 413 K (phase I) with structural snapshots at energy maxima and minima. The in-plane rotation of the Im cation is set in steps of 10° over 360°. Note: the host channel is set rigid and only the in-plane rotation of the Im cation is scanned.

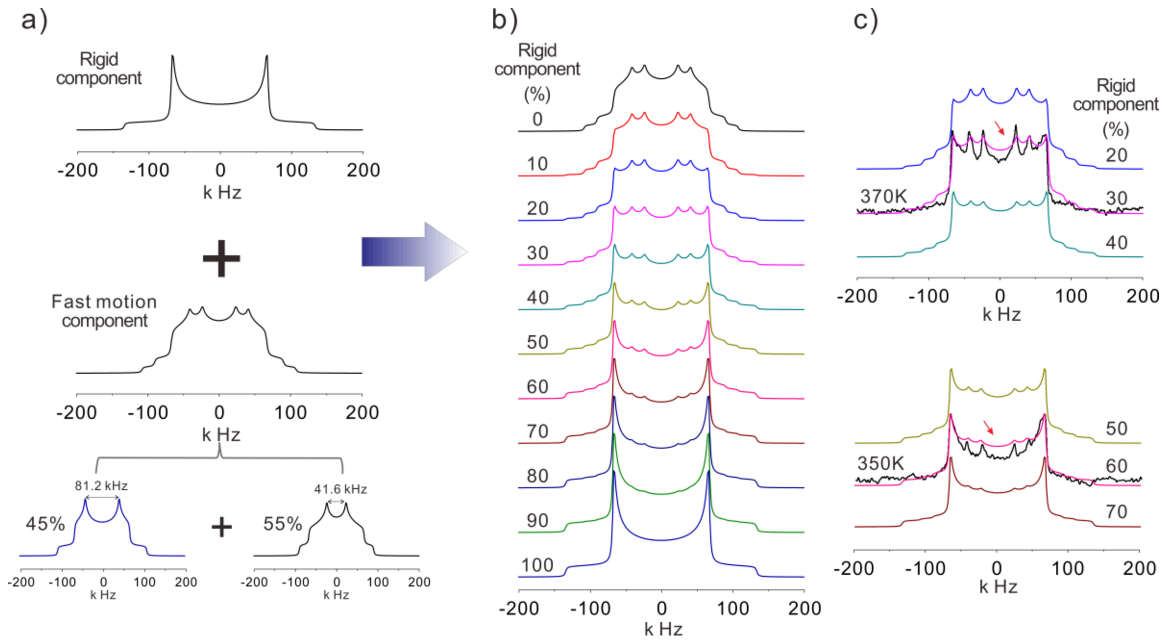

Supplementary Figure 15 | Simulation of the ^{2}H patterns. (**a**) The procedure to obtain the simulated patterns in (**b**). In the first summation, the two patterns are simulated based on the in-plane jump motion model in the fast limit with the jump angles of 55.5° and 42.1°. The component ratio A:B is 45:55. (**b**) The summed patterns, showing the lineshape change upon the change in the content of the rigid component. (**c**) The comparison between the experimental patterns acquired at 350 K and 370 K (the black lines) and the simulated patterns. We also simulated the patterns based on the similar in-plane jump motion in the fast limit but with the different jump angles, i.e., (55.5°, 84.3°, 95.7° and 124.5°) and (42.1° and 137.9°), which can reproduce the horns having the splitting of 81.2 kHz and 41.6 kHz, respectively. But the results clearly show that the simulated patterns do not fit the experimental ones.

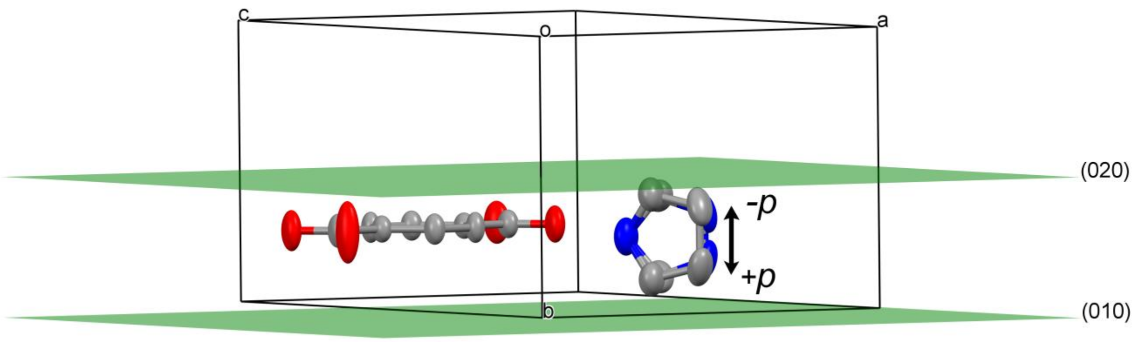

Supplementary Figure 16 | Schematic illustration of the contribution of the reorientation of the Im cation to the induced dipole moments (*p*) in 1-d_0 at 413 K. There is a relationship between the microscopic quantity *p* and the macroscopic quantity *ε*' for an ideal non-interactive systems, $(\varepsilon' - 1)\varepsilon_0 E = \Sigma p/V$, where ε_0 is the vacuum permittivity, *E* is the electric field and *V* is the volume. In addition, the nonzero *b* and zero *a* and *c* components of the *p* explain well the experimentally observed dielectric anisotropy.

Supplementary Table 1 | Selected crystallographic data and structural refinement details for 1.

Compound	**1-d$_0$**	**1-d$_0$**	**1-d$_0$**	**1-d$_3$**	**1-d$_3$**	**1-d$_3$**
Temperature / K	293(2)	363(2)	413(2)	293(2)	363(2)	413(2)
Formula	$C_{11}H_{10}N_2O_4$	$C_{11}H_{10}N_2O_4$	$C_{11}H_{10}N_2O_4$	$C_{11}H_7D_3N_2O_4$	$C_{11}H_7D_3N_2O_4$	$C_{11}H_7D_3N_2O_4$
Crystal size / mm	0.24×0.22×0.20	0.24×0.22×0.20	0.24×0.22×0.20	0.20×0.20×0.20	0.20×0.20×0.20	0.20×0.20×0.20
Formula weight	234.21	234.21	234.21	237.22	237.22	237.22
Crystal system	Monoclinic	Monoclinic	Orthorhombic	Monoclinic	Monoclinic	Orthorhombic
Space group	$P2_1/c$	$P2_1/c$	*Pnma*	$P2_1/c$	$P2_1/c$	*Pnma*
a / Å	6.980(5)	7.053(5)	9.670(9)	6.981(4)	7.054(5)	9.672(7)
b / Å	15.977(10)	15.941(11)	7.112(7)	15.958(10)	15.938(11)	7.100(5)
c / Å	9.641(6)	9.664(7)	15.869(15)	9.640(6)	9.657(6)	15.865(12)
α / °	90.00	90.00	90.00	90.00	90.00	90.00
β / °	95.398(12)	94.962(12)	90.00	95.379(13)	95.076(16)	90.00
γ / °	90.00	90.00	90.00	90.00	90.00	90.00
Volume / $Å^3$	1070(1)	1083(1)	1091(2)	1069(1)	1082(1)	1090(1)
Z	4	4	4	4	4	4
D_{calc} / g·cm^{-3}	1.453	1.437	1.425	1.474	1.457	1.446
μ / mm^{-1}	0.113	0.112	0.111	0.113	0.112	0.111
F(000)	488	488	488	488	488	488
θ range / °	2.48–27.45	3.67–27.48	3.14–27.45	3.20–27.47	3.17–27.43	3.78–27.49
Reflns. collected	11391	11483	11076	11355	11364	11178
Indep. reflns. (R_{int})	2434 (0.0321)	2460 (0.0431)	1345 (0.0488)	2442 (0.0437)	2457 (0.0383)	1349 (0.0479)
Obsd. reflns. [I>2σ(I)]	2066	1842	1125	2036	1958	1120
Parameters	157	158	118	158	158	118
R_1^a/wR_2^b [I>2σ(I)]	0.0447/0.1148	0.0550/0.1292	0.0704/0.1741	0.0489/0.1270	0.0610/0.1519	0.0773/0.1688
R_1/wR_2 (all data)	0.0538/0.1213	0.0771/0.1409	0.0850/0.1853	0.0608/0.1355	0.0768/0.1610	0.0926/0.1774
GOF	1.083	1.075	1.201	1.107	1.155	1.251
$\Delta\rho^c$ / e·Å^{-3}	0.248/−0.208	0.200/−0.198	0.232/−0.218	0.253/−0.187	0.254/−0.195	0.233/−0.164

Compound	**1-d$_2$**	**1-d$_2$**	**1-d$_2$**	**1-d$_5$**	**1-d$_5$**	**1-d$_5$**
Temperature / K	293(2)	363(2)	413(2)	293(2)	363(2)	413(2)
Formula	$C_{11}H_7D_3N_2O_4$	$C_{11}H_7D_3N_2O_4$	$C_{11}H_7D_3N_2O_4$	$C_{11}H_4D_6N_2O_4$	$C_{11}H_4D_6N_2O_4$	$C_{11}H_4D_6N_2O_4$
Crystal size / mm	0.20×0.20×0.20	0.20×0.20×0.20	0.20×0.20×0.20	0.20×0.20×0.20	0.20×0.20×0.20	0.20×0.20×0.20
Formula weight	237.22	237.22	237.22	240.22	240.22	240.22
Crystal system	Monoclinic	Monoclinic	Orthorhombic	Monoclinic	Monoclinic	Orthorhombic
Space group	$P2_1/n$	$P2_1/c$	*Pnma*	$P2_1/n$	$P2_1/c$	*Pnma*
a / Å	9.768(5)	7.051(6)	9.695(6)	9.759(4)	7.073(8)	9.696(9)
b / Å	15.869(9)	15.889(13)	7.123(4)	15.864(7)	15.884(17)	7.116(7)
c / Å	13.867(8)	9.677(8)	15.862(9)	13.840(6)	9.688(10)	15.873(14)
α / °	90.00	90.00	90.00	90.00	90.00	90.00
β / °	90.345(10)	92.930(17)	90.00	90.339(8)	93.04(3)	90.00
γ / °	90.00	90.00	90.00	90.00	90.00	90.00
Volume / Å^3	2149(2)	1083(2)	1095(1)	2143(2)	1087(2)	1095(2)
Z	8	4	4	8	4	4
D_{calc} / g·cm^{-3}	1.466	1.455	1.438	1.489	1.468	1.457
μ / mm^{-1}	0.112	0.112	0.110	0.113	0.111	0.110
F(000)	976	488	488	976	488	488
θ range / °	3.61–27.46	2.89–27.48	3.13–27.43	2.55–27.49	3.71–27.45	3.78–27.46
Reflns. collected	23138	11376	11335	23038	11302	11263
Indep. reflns. (R_{int})	4887 (0.0475)	2462 (0.0477)	1350 (0.0493)	4884 (0.0373)	2459 (0.0464)	1353 (0.0485)
Obsd. reflns. [I>2σ(I)]	3915	1792	1034	4142	1853	1114
Parameters	315	158	118	315	158	118
R_1^a/wR_2^b [I>2σ(I)]	0.0539/0.1225	0.0640/0.1609	0.0687/0.1598	0.0479/0.1173	0.0610/0.1567	0.0721/0.1639
R_1/wR_2 (all data)	0.0717/0.1318	0.0895/0.1783	0.0912/0.1735	0.0584/0.1234	0.0827/0.1730	0.0895/0.1746
GOF	1.104	1.099	1.171	1.091	1.101	1.222
$\Delta\rho^c$ / e·Å^{-3}	0.266/−0.225	0.432/−0.272	0.203/−0.157	0.280/−0.217	0.443/−0.236	0.262/−0.165

^{a} $R_F = \Sigma||F_o| - |F_c||/\Sigma|F_o|$. ^{b} $wR_2(F^2) = [\Sigma w(F_o^2 - F_c^2)^2/\Sigma wF_o^4]^{1/2}$. ^{c} Maximum and minimum residual electron density.

Supplementary Table 2 | Selected hydrogen bonds (Å, °) for 1.

	D−H···A	D−H / Å	H···A / Å	D···A / Å	∠DHA / °
**1-d$_0$** (293 K)	N1−H1···O2^{i}	0.86	1.85	2.675(2)	161
	N2−H2···O4^{ii}	0.86	2.01	2.847(2)	166
	O3−H1A···O1^{iii}	1.10(3)	1.37(3)	2.469(2)	179(2)
	Symmetry codes: (i) −*x*+1, −*y*, −*z*; (ii) *x*+1, −*y*+1/2, *z*−1/2; (iii) *x*, *y*, *z*+1.				
**1-d$_0$** (363 K)	N1−H1···O2^{i}	0.86	1.87	2.693(3)	161
	N2−H2···O4^{ii}	0.86	2.05	2.892(3)	165
	O3−H1A···O1^{iii}	1.14(3)	1.34(3)	2.478(2)	177(2)
	Symmetry codes: (i) −*x*+1, −*y*, −*z*; (ii) *x*+1, −*y*+1/2, *z*−1/2; (iii) *x*, *y*, *z*+1.				
**1-d$_0$** (413 K)	N1−H1···O2^{ii}	0.93	1.84	2.712(4)	156
	O3−H1A···O1^{iii}	1.09(6)	1.40(6)	2.492(3)	178(5)
	Symmetry codes: (ii) −*x*, −*y*+1, −*z*; (iii) *x*+1, *y*, *z*.				

	D−H···A	D−H / Å	H···A / Å	D···A / Å	∠DHA / °
**1-d$_3$** (293 K)	N1−H1···O2^{i}	0.86	1.85	2.676(2)	161
	N2−H2···O4^{ii}	0.86	2.01	2.847(2)	166
	O3−H1A···O1^{iii}	1.15(3)	1.32(3)	2.467(2)	178(2)
	Symmetry codes: (i) −*x*+1, −*y*, −*z*; (ii) *x*+1, −*y*+1/2, *z*−1/2; (iii) *x*, *y*, *z*+1.				
**1-d$_3$** (363 K)	N1−H1···O2^{i}	0.86	1.86	2.692(3)	161
	N2−H2···O4^{ii}	0.86	2.05	2.890(3)	165
	O3−H1A···O1^{iii}	1.16(4)	1.31(4)	2.475(2)	179(3)
	Symmetry codes: (i) −*x*+1, −*y*, −*z*; (ii) *x*+1, −*y*+1/2, *z*−1/2; (iii) *x*, *y*, *z*+1.				
**1-d$_3$** (413 K)	N1−H1···O2^{ii}	0.93	1.84	2.711(4)	156
	O3−H1A···O1^{iii}	1.10(6)	1.39(6)	2.491(3)	179(5)
	Symmetry codes: (ii) −*x*, −*y*+1, −*z*; (iii) *x*+1, *y*, *z*.				

	D−H···A	D−H / Å	H···A / Å	D···A / Å	∠DHA / °
**1-d₂** (293 K)	N3−D3A···O2^{i}	0.86	1.98	2.831(2)	172
	N4−D4A···O6^{ii}	0.86	1.83	2.672(2)	167
	N2−D2···O5^{ii}	0.86	1.88	2.719(2)	165
	N1−D1···O2	0.86	1.96	2.775(2)	159
	O3−D1A···O1^{iii}	0.99(3)	1.58(3)	2.554(2)	171(3)
	O7−D2A···O5^{ii}	1.03(3)	1.56(3)	2.590(2)	178(2)
	Symmetry codes: (i) −*x*+1/2, *y*−1/2, −*z*+1/2; (ii) *x*+1, *y*, *z*; (iii) *x*−1, *y*, *z*.				
**1-d₂** (363 K)	N1−D1···O2^{i}	0.86	1.88	2.706(3)	161
	N2−D2···O4^{ii}	0.86	2.17	2.997(4)	161
	O3−D1A···O1^{iii}	1.07(4)	1.43(4)	2.498(3)	179(3)
	Symmetry codes: (i) −*x*+1, −*y*, −*z*; (ii) *x*+1, −*y*+1/2, *z*−1/2; (iii) *x*, *y*, *z*+1.				
**1-d₂** (413 K)	N1−D1···O2^{ii}	0.93	1.85	2.724(4)	156
	O3−D1A···O1^{iii}	1.19(6)	1.32(6)	2.510(3)	179(4)
	Symmetry codes: (ii) −*x*, −*y*+1, −*z*; (iii) *x*+1, *y*, *z*.				

	D−H···A	D−H / Å	H···A / Å	D···A / Å	∠DHA / °
**1-d₅** (293 K)	N3−D3A···O2^{i}	0.86	1.97	2.829(2)	172
	N4−D4A···O6^{ii}	0.86	1.83	2.669(2)	166
	N2−D2···O5^{ii}	0.86	1.88	2.715(2)	165
	N1−D1···O2	0.86	1.95	2.771(2)	158
	O3−D1A···O1^{iii}	0.95(3)	1.61(3)	2.551(2)	170(2)
	O7−D2A···O5^{ii}	0.98(3)	1.61(3)	2.590(2)	178(2)
	Symmetry codes: (i) −*x*+1/2, *y*−1/2, −*z*+1/2; (ii) *x*+1, *y*, *z*; (iii) *x*−1, *y*, *z*.				
**1-d₅** (363 K)	N1−D1···O2^{i}	0.86	1.88	2.706(3)	161
	N2−D2···O4^{ii}	0.86	2.17	2.993(4)	161
	O3−D1A···O1^{iii}	1.06(3)	1.44(3)	2.498(3)	177(3)
	Symmetry codes: (i) −*x*+1, −*y*, −*z*; (ii) *x*+1, −*y*+1/2, *z*−1/2; (iii) *x*, *y*, *z*+1.				
**1-d₅** (413 K)	N1−D1···O2^{ii}	0.93	1.85	2.726(4)	157
	O3−D1A···O1^{iii}	1.17(6)	1.34(6)	2.509(4)	179(4)
	Symmetry codes: (ii) −*x*, −*y*+1, −*z*; (iii) *x*+1, *y*, *z*.				

Supplementary Table 3 | $\pi\cdots\pi$ interactions between neighboring benzene rings in 1-d$_0$ and 1-d$_5$, showing a typical OFF (offset face-to-face) motif[1].

Compound	Temperature (phase)	$d_{centroid}$ / Å [a]	d_{plane} / Å [b]	d_{offset} / Å [c]	ϕ / ° [d]
**1-d$_0$**	293 K (II)	3.845	3.464	1.668	0.76
	363 K (II)	3.890	3.509	1.679	0.70
	413 K (I)	3.930	3.556	1.673	0
**1-d$_5$**	363 K (II)	3.903	3.528	1.669	0.56
	413 K (I)	3.938	3.558	1.688	0

[a] $d_{centroid}$: centroid⋯centroid distance of two rings; [b] d_{plane}: distance between centroid and ring plane; [c] d_{offset}: distance given by $(d_{centroid}^2 - d_{plane}^2)^{1/2}$, reflecting the degree of overlap of the two rings; [d] ϕ: dihedral angle between the two ring planes.

Supplementary Table 4 | Parameters used to simulate the patterns in Figure 6a.

Temperature (K)	Quadruplar coupling (kHz)	Rigid component	Jump rate (kHz)	Width of jump rate distribution (lgGauss)
300	134	15%	25	1
310	134	15%	55	1
330	134	15%	150	1
350	134	15%	350	1
360	134	10%	500	1
370	134	10%	700	1
380	134	5%	100	1
390	134	5%	1500	2
400	132	3%	2500	2
420	132	2%	4000	3
430	132	2%	6000	3
450	132	2%	10000	3

Supplementary References

1. Russell, V., Scudder, M., Dance I. The crystal supramolecularity of metal phenanthroline complexes. *J. Chem. Soc., Dalton Trans.* **6**, 789−799 (2001).
